# Upregulation of homeobox gene is correlated with poor survival outcomes in cervical cancer

**DOI:** 10.18632/oncotarget.21041

**Published:** 2017-09-16

**Authors:** Kyung Jin Eoh, Hee Jung Kim, Jung-Yun Lee, Eun Ji Nam, Sunghoon Kim, Sang Wun Kim, Young Tae Kim

**Affiliations:** ^1^ Institute of Women's Medical Life Science, Department of Obstetrics and Gynecology, Yonsei University College of Medicine, Seoul, Korea

**Keywords:** cervical cancer, homeobox genes, survival, TCGA, biomarker

## Abstract

*HOX* family members encode transcription factors crucial for embryogenesis and may be associated with carcinogenesis. Here, we evaluated the expression of 39 *HOX* genes in cervical cancer by using clinicopathological information and gene expression data of 308 patients from The Cancer Genome Atlas (TCGA) database. Correlations between mRNA expression of HOX family members and clinicopathological variables were explored. Seventy-three (23.7%) patients died during the follow-up period (median, 22.0 months). Overall mortality was significantly associated with advanced FIGO stage, lymph node metastasis, lymphovascular invasion, and increased *HOXA1*, *HOXA5*, *HOXA6*, and *HOXC11* mRNA expression. Kaplan–Meier survival analysis revealed that overall survival was significantly shorter in patients with high *HOXA* rather than low *HOXA* expression (*HOXA1*, P = 0.012; *HOXA5*, P = 0.008; and *HOXA6*, P = 0.006). Upregulated *HOXA1*, *HOXA5*, and *HOXA6* expression are significantly correlated with unfavorable overall survival and increased mortality in cervical cancer patients. Therefore, *HOXA* expression is a potential cervical cancer prognostic indicator.

## INTRODUCTION

Cervical cancer is a multifactorial disease caused due to genetic, environmental, and epigenetic factors, as well as infection by human papillomavirus [1]. Recently, alterations in the expression of transcription factors have been focused upon for its importance in the development of this malignancy.

A large amount of scientific evidence indicates that the expression levels of many genes involved in normal embryo development are aberrant in and contribute to carcinogenesis [2]. *Homeobox (HOX)* genes encode homeoproteins, which function as transcription factors in the differentiation and proliferation processes at the time of development of embryonic structures, and their aberrant expression has been found to be associated with carcinogenesis and aggressiveness [3, 4]. *HOX* genes were first described as factors involved in embryogenesis in the fruit fly, *Drosophila melanogaster* [5]. *HOX* genes commonly share a 120-base pair DNA sequence called the homeobox, which codes a 61-amino acid peptide termed as the homeodomain. This domain has been characterized previously using nuclear magnetic resonance spectroscopy [6, 7]. *HOX* genes and their protein structures have also been found in humans [8, 9]. In the human genome, there are 39 *HOX* genes located on four different chromosomes, and these genes are the structural and functional homologs of the homeotic complex of *Drosophila* [10].

Previous studies have reported that upregulation of the *HOX* gene is related to adverse prognostic factors in cervical cancer, but the majority of these studies have been limited by small sample sizes and have been performed only *in vitro*. Aberrant expression of *HOXD9*, *HOXC5*, *HOXC8*, and *HOXB2, HOXB4, HOXC10*, and *HOXD13* has been found in cervical cancer cell lines [11–13]. A recent study focused on the mechanism of HPV16 E7-mediated epigenetic regulation of *HOX* genes showed the master regulatory role of HPV16 E7 in modulating the expression of *HOX* cluster genes [14]. Several studies have also revealed the potential roles of *HOX antisense* long non-coding RNAs in cervical cancer aggressiveness [15–17]. These results suggest that aberrant expression of *HOX*-related genes is associated with the process of cervical carcinogenesis.

Therefore, in this study, we aimed to analyze the mRNA expression levels of all the 39 *HOX* genes using data from The Cancer Genome Atlas (TCGA) database [18]. We then went on to explore their correlations with clinical data including survival outcomes in cervical cancer and evaluated the prognostic value of *HOX* gene expression analysis. To the best of our knowledge, this is the first study to analyze the expression of all *HOX* genes using data from the TCGA database. Our findings indicate that measurement of *HOX* gene expression can help predict prognosis and overall survival in cervical cancer.

## RESULTS

A total of 308 cervical cancer cases were included in this study; of these, 253 were squamous cell carcinoma, 28 adenocarcinomas, 17 mucinous carcinomas, three endometrioid carcinomas, and seven adenosquamous carcinoma cases. The median follow-up period was 22.0 months (range: 0.1–213.6 months). Among the clinicopathological features, overall mortality was found to be associated with FIGO stage III/IV, lymph node metastasis, and lymphovascular invasion (Table 1).

**Table 1:** Correlation between clinicopathological features and overall mortality

Variables	Dead, n=73 (%)	Alive, n=235 (%)	P value
Age			
<45	26 (35.6)	110 (46.8)	0.093
≥45	47 (64.4)	125 (53.2)	
Stage			
I/II	48 (65.8)	185 (81.5)	**0.005**
III/IV	25 (34.2)	42 (18.5)	
Histology			
Squamous Cell	62 (84.9)	191 (81.6)	0.733
Adenocarcinoma	5 (6.8)	23 (9.8)	
Mucinous	5 (6.8)	12 (5.1)	
Endometrioid	0	3 (1.3)	
Adenosquamous	1 (1.4)	5 (2.1)	
Grade			
1, 2	40 (63.5)	115 (54.0)	0.182
3, 4	23 (36.5)	98 (46.0)	
ECOG			
0,1	39 (90.7)	156 (95.7)	0.246
2,3	4 (9.3)	7 (4.3)	
LN metastasis			
Yes	19 (54.3)	35 (27.3)	**0.003**
No	16 (45.7)	93 (72.7)	
LVI			
Yes	25 (92.6)	58 (45.3)	**<0.001**
No	2 (7.4)	70 (54.7)	

ECOG, The Eastern Cooperative Oncology Group performance score; LN, lymph node; LVI, lymphovascular invasion.

Table 2 shows the correlations between the clinicopathological features and the mRNA expression levels of *HOX* family genes. When the cases were categorized into low and high mRNA expression groups based on cut-off values determined as medians for each gene, higher *HOXA1*, *HOXA5*, *HOXA6*, and *HOXC11* expression was found to be associated with increased overall mortality (odds ratio (OR): 1.858, 95% confidence interval (CI): 1.086–3.178; OR: 2.003, 95% CI: 1.167–3.438; OR: 2.162, 95% CI: 1.256–3.724; and OR: 1.724, 95% CI: 1.011–2.942, respectively). High FIGO stage also highly correlated with high *HOXB7* expression, and lymph node metastasis was found to be associated with high *HOXD12* and *HOXD13* expression (P < 0.01). Moreover, squamous cell histologic type was found to be highly correlated with high expression of *HOXA1*, *HOXA4*, *HOXA7*, *HOXA9*, *HOXB2*, *HOXB3*, *HOXB5–9*, *HOXC4*, *HOXC5*, *HOXC13*, *HOXD1*, *HOXD9*, *HOXD10*, *HOXD11*, and *HOXD13* (P < 0.001).

**Table 2:** Summary of the correlation between clinicopathological features and mRNA expression counts of homeobox (*HOX*) family genes

	Age≥45	Stage III/IV	Squamous cell	Grade3/4	ECOG2/3	LN metastasis	LVI	Mortality
*HOXA1*			+++					+
*HOXA2*				+		+		
*HOXA3*								
*HOXA4*			+++					
*HOXA5*			++					++
*HOXA6*				+				++
*HOXA7*			+++					
*HOXA9*			+++					
*HOXA10*								
*HOXA11*				+				
*HOXA13*								
*HOXB1*				+				
*HOXB2*			+++					
*HOXB3*		+	+++					
*HOXB4*			++					
*HOXB5*			+++			+		
*HOXB6*			+++		+			
*HOXB7*		++	+++					
*HOXB8*		+	+++					
*HOXB9*		+	+++			+		
*HOXB13*			+					
*HOXC4*			+++					
*HOXC5*	++		+++					
*HOXC6*	++		+					
*HOXC8*	++		+					
*HOXC9*								
*HOXC10*			++					
*HOXC11*	+		+			+		+
*HOXC12*								
*HOXC13*			+++					
*HOXD1*			+++					
*HOXD3*			++				+	
*HOXD4*			+					
*HOXD8*			+		+			
*HOXD9*		+	+++			+	+	
*HOXD10*			+++				+	
*HOXD11*			+++			+		
*HOXD12*			+			++		
*HOXD13*			+++			++	+	

+, correlation with P value < 0.05; ++, correlation with P value <0.01; +++, correlation with P value < 0.001; ECOG, The Eastern Cooperative Oncology Group performance score; LN, lymph node; LVI, lymphovascular invasion.

Linear regression analysis indicated that overall mortality was significantly associated with high mRNA expression levels of *HOXA1* (t = 3.033, P = 0.003) (Table 3). High expression of the *HOXA1* gene was also found to be positively correlated with squamous cell histologic type and lymph node metastasis.

**Table 3:** Linear regression analysis between clinicopathological features and mRNA expression count of *HOXA1*, *HOXA5*, *HOXA6*, and *HOXC11*

	*HOXA1*		*HOXA5*		*HOXA6*		*HOXC11*	
	t value	P value	t value	P value	t value	P value	t value	P value
Age≥45	-1.379	0.172	0.081	0.936	-0.822	0.413	0.477	0.635
Stage III/IV	-0.927	0.357	0.176	0.861	-0.066	0.948	-0.412	0.682
Squamous cell	4.536	**<0.001**	1.718	**0.09**	-0.138	0.891	-2.94	**0.004**
Grade ¾	1.567	0.121	-0.076	0.94	-0.917	0.362	-1.099	0.275
ECOG 2/3	0.358	0.721	1.458	0.149	1.585	0.117	-0.449	0.655
LN metastasis	1.907	**0.06**	-0.233	0.816	0.931	0.355	0.675	0.501
LVI	-1.403	0.165	-0.828	0.41	-0.12	0.905	0.137	0.891
Mortality	3.033	**0.003**	0.709	0.48	1.209	0.23	-0.23	0.819

ECOG, The Eastern Cooperative Oncology Group performance score; LN, lymph node; LVI, lymphovascular invasion.

**Figure 1:**
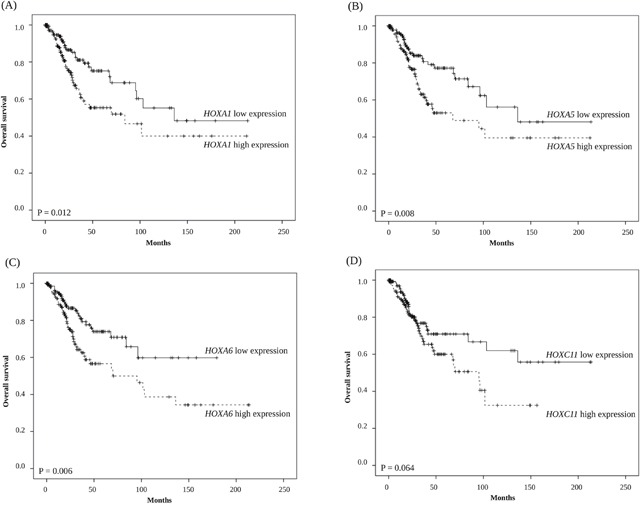
Kaplan-Meier survival analysis of 308 cervical cancer patients stratified by **(A)** *HOXA1*, **(B)** *HOXA5*, **(C)** *HOXA6*, and **(D)** *HOXC11* gene expression levels.

In Kaplan–Meier survival analyses, we found that groups with higher *HOXA1*, *HOXA5*, and *HOXA6* mRNA expression levels had significantly unfavorable overall survival than those with a lower level of expression of these genes (P = 0.012, P = 0.008, and P = 0.006, respectively) (Figure 1).

## DISCUSSION

In this study, we showed that the increased mRNA expression of four HOX genes—*HOXA1*, *HOXA5*, *HOXA6*, and *HOXC11*—is independently associated with mortality in cervical cancer based on data from TCGA [18]. Several *HOX* genes were shown to be correlated with risk factors for poor prognoses including advanced FIGO stage, high grade, lymph node metastasis, and lymphovascular invasion. Interestingly, high expression of most of the *HOX* genes was found to be strongly associated with squamous cell type histology. In particular, the upregulation of the *HOXA1*, *HOXA5*, and *HOXA6* genes were found to be significantly associated with unfavorable overall survival in patients with cervical cancer.

Cancers have been revealed as aberrations in the growth, differentiation, and organization of cell populations [19]. These basic processes are supposed to be tightly coordinated and controlled during embryogenesis as well as in adult tissues [20]. The oncogerminative theory of cancer development suggests that malignant transformation occurs due to the aberrant expression of development-related genes [21]. According to this concept, carcinogenesis is a dynamic self-organizing process that resembles the process of early embryo development. The malignant transformation that arises from gene mutations in combination with epigenetic dysregulation eventually results in reprogramming of somatic cells into immortal cells that simulate germline cells, which is consistent with the characteristics of cancer stem cells or, in other words, oncogerminative cells.

As a developing malignant transformation, the oncogerminative cell is considered to follow the same biological principles as those in play in a germline cell as it develops into a blastocyst-stage embryo [21]. The oncogerminative theory of cancer development hypothesizes that to have a cancer is the same as gestating a damaging embryo. This oncogerminative hypothesis of tumor growth includes five stages of tumor development: (i) malignant transformation of normal cells into oncogerminative cells, i.e. cancer stem cells; (ii) reproduction of the oncogerminative cells; (iii) formation of a multicellular spheroid (a parody of blastocyst formation) characterized by a heterogeneous population of cells in addition to the oncogerminative cells; (iv) vascularization of the oncospheroid and its growth; and (v) development of the majority of malignant tumors together with disaggregation of the oncogerminative cells, their migration into the organism's tissues, and development of metastatic foci of tumor growth [22].

A considerable body of evidence has proven the crucial role of *HOX* genes as developmental genes during embryogenesis as well as the critical role of aberrant *HOX* gene expression in the development of various tumors [2–4]. However, to the best of our knowledge, our study is the first one to be based on TCGA data and reporting the correlation between upregulated *HOX* gene expression and aggressiveness of cervical carcinoma. Nevertheless, further studies are needed in the future to fully understand the role of *HOX* genes in cervical cancer.

Our results also indicate that the analysis of *HOX* gene expression, especially in cytological or surgical specimens, can help identify patients with cervical cancer who are expected to have a poor prognosis. According to the results of the analysis, we can then recommend more aggressive treatments or more frequent follow-ups for such cases with high risk. Furthermore, novel therapeutic agents need to be developed for refractory cervical cancer patients administered standard treatment. Our results highlighting the correlation between *HOX* gene upregulation and poor survival outcomes indicate that drugs inhibiting *HOXA* gene expression could have the ability to induce the inhibition of oncogerminative cells and thus help in the treatment of refractory cervical cancer patients. In fact, one previous *in vitro* study has shown the potential of the use of a homeobox transcription factor inhibitor in this respect [23].

One major limitation of this study is the short-term follow-up period; the small number of mortalities observed during this period might weaken the clinical applicability of the current findings. A more comprehensive investigation based on regularly updated TCGA data is required in the future. Nevertheless, this study proved the hypothesis that overexpression of *HOXA* genes is associated with poor prognosis in cervical cancer, based on data obtained using the RNA-seq technique. However, further validation using a different modality such as a microarray or immunohistochemical assay with a large volume might also be needed to verify the results of the present study.

In conclusion, in this study, we showed that the upregulation of the expression of the *HOXA1*, *HOXA5*, and *HOXA6* genes are significantly associated with unfavorable overall survival as well as increased mortality in a large cohort of cervical cancer cases from TCGA database. Our results indicate that the evaluation of *HOXA* gene expression may be valuable for predicting prognosis in cervical carcinoma. Also, we could consider *HOX* gene expression levels may have a potential for use as biomarkers for the same purpose.

## MATERIALS AND METHODS

### Data acquisition

We obtained mRNA expression counts for 39 *HOX* family genes and the corresponding clinicopathological information from the TCGA data portal (https://tcga-data.nci.nih.gov/tcga/tcgaDownload.jsp). The personal information of the patients is anonymized, and the patients were deidentified. According to TCGA publication guidelines (http://cancergenome.nih.gov/publications/publicationguidelines), these mRNA sequencing data have no restrictions on publication, and no additional approval by an ethics committee was required to publish the use of the data.

The Illumina Genome Analyzer was utilized as the platform for DNA sequencing (Illumina Inc, San Diego, CA). RNA sequencing data were obtained using Illumina HiSeq 2000 RNA Sequencing Version 2 analysis, and the mRNA expression counts were expressed as RNA-Seq data normalized to data from the pan-cancer database.

### Patients

We collected mRNA expression data for 39 *HOX* family genes for each patient, along with their clinicopathological features including age at initial pathologic diagnosis, FIGO (The International Federation of Gynecology and Obstetrics) stage, histologic subtype, ECOG (Eastern Cooperative Oncology Group) performance score, lymph node metastasis, lymphovascular invasion, and overall survival (Supplementary Table 1).

### Statistical analyses

We used the Fisher exact or χ^2^ tests for categorical variables according to sample size. Linear regression was applied to assess the associations between clinical variables and the expression counts of each *HOX* gene. We also evaluated t values for correlation coefficients. Median of *HOX* gene expression was determined as a cut-off value for the prediction of survival. Kaplan-Meier survival analyses based on calculated cut-off values were performed. SPSS version 23.0 (IBM Corp., Armonk, NY, USA) was used for data analysis.

## SUPPLEMENTARY MATERIALS FIGURES AND TABLES




