# Supplementary material for: Upregulation of homeobox gene is correlated with poor survival outcomes in cervical cancer
Source: Oncotarget. 2017 Sep 16;8(48):84396–402. doi: 10.18632/oncotarget.21041 (PMC5663605; doi:10.18632/oncotarget.21041)
Supplement: Supplementary file 1 [file oncotarget-08-84396-s001.pdf]

Upregulation of homeobox gene is correlated with poor survival outcomes in cervical cancer

SUPPLEMENTARY MATERIAL

Supplementary Table 1: Patient clinicopathologic information with mRNA expression levels of HOX family genes

See Supplementary File 1
